# Attitudes and Practices of Immune Checkpoint Inhibitors in Chinese Patients With Cancer: A National Cross-Sectional Survey

**DOI:** 10.3389/fphar.2021.583126

**Published:** 2021-03-22

**Authors:** Luping Zhang, Jun Wang, Bicheng Zhang, Qian Chu, Chunxia Su, Hao Wu, Xiaobing Chen, Baocheng Wang, Yongmei Yin, Bo Zhu, Jianguo Sun

**Affiliations:** ^1^ Cancer Institute, Xinqiao Hospital, Army Medical University, Chongqing, China; ^2^ Department of Oncology, The First Affiliated Hospital of Shandong First Medical University, Jinan, China; ^3^ Cancer Center, Renmin Hospital of Wuhan University, Wuhan, China; ^4^ Department of Oncology, Tongji Hospital, Tongji Medical College, Huazhong University of Science and Technology, Wuhan, China; ^5^ Associate Chief Physician of Medical Oncology, Shanghai Pulmonary Hospital and Thoracic Cancer Institute, Tongji University School of Medicine, Shanghai, China; ^6^ Department of Oncology, The First Affiliated Hospital of Nanjing Medical University, Nanjing, China; ^7^ Department of Oncology, The Affiliated Cancer Hospital of Zhengzhou University, Henan Cancer Hospital, Zhengzhou, China; ^8^ Department of Oncology, No. 960 Hospital of PLA, Jinan, China

**Keywords:** immunotherapy, adverse effects, attitude, practice, survey

## Abstract

Immune-checkpoint inhibitors (ICIs) are revolutionizing the field of immuno-oncology. Side effects and tumor microenvironment currently represent the most significant obstacles to using ICIs. In this study, we conducted an extensive cross-sectional survey to investigate the concept and practices regarding the use of ICIs in cancer patients in China. The results provide real-world data on the adverse events (AEs) of ICIs and the factors influencing the use of ICIs. This survey was developed by the Expert Committee on Immuno-Oncology of the Chinese Society of Clinical Oncology (CSCO-IO) and the Expert Committee on Patient Education of the Chinese Society of Clinical Oncology (CSCO-PE). The surveys were distributed using a web-based platform between November 29, 2019 and December 21, 2019. A total of 1,575 patients were included. High costs (43.9%), uncertainty about drug efficacy (41.2%), and no reimbursement from medical insurance (32.4%) were the factors that prevented the patients from using ICIs. The patients were most concerned about the onset time or effective duration of ICIs (40.3%), followed by the indications of ICIs and pre-use evaluation (33.4%). Moreover, 9.0, 57.1, 21.0, and 12.9% of the patients reported tumor disappearance, tumor volume reduction, no change in tumor volume, and increased tumor volume. Among the patients who received ICIs, 65.7% reported immune-related AEs (irAEs); 96.1% reported mild-to-moderate irAEs. Cancer patients in China had a preliminary understanding of ICIs. Yet, the number of patients treated with ICIs was small.

## Introduction

Immune checkpoint inhibitors (ICIs) are a type of anticancer therapy that acts by suppressing immune inhibitory pathways such as the cytotoxic lymphocytes antigen proteins (CTLA-4) pathway and the programmed cell death protein-1 (PD-1)/programmed death-ligand-1 (PD-L1) axis (Pardoll, 2012). Cancer cells can activate those pathways to evade immune surveillance, but when checkpoints are blocked, the immune cells can kill cancer cells (Cameron et al., 2011; Karwacz et al., 2011; Pardoll, 2012). Recently, some ICIs have been approved by the FDA, including ipilimumab that targets CTLA4 (Cameron et al., 2011); nivolumab, pembrolizumab, and cemiplimab, which target PD-1 (Rajan et al., 2016; Vachhani and Chen, 2016; Migden et al., 2020); atezolizumab, avelumab, and durvalumab that can block the PD-L1 axis (Kaufman et al., 2018; Schmid et al., 2018; Senan et al., 2019). Moreover, China has recently launched a few new ICIs, including camrelizumab (Wei et al., 2019), toripalimab (Song et al., 2020), and tisleizumab (Wang et al., 2019), which have been approved for the treatment of Hodgkin’s lymphoma.

ICIs have shown high efficacy in treating certain types of cancer. For example, the pooled hazard ratios (HRs) for overall survival (OS) and progression-free survival (PFS) in lung cancer patients treated with PD-1/PD-L1 inhibitors were 0.69 and 0.74, respectively, while the odds ratios (ORs) for treatment-related grade 3–5 adverse events (AEs) were 0.30–0.33 (Lai et al., 2020). Similar results were observed in the treatment of classic Hodgkin’s lymphoma (Zhang et al., 2019), renal cell carcinoma (Sun et al., 2020), urothelial cancer (Niglio et al., 2019), melanoma (Karlsson and Saleh, 2017), and breast cancer (Vranic et al., 2019). The NCCN (NCCN, 2019a; NCCN, 2019b; NCCN, 2020a; NCCN, 2020b), ESMO (Dummer et al., 2015; Escudier et al., 2016; Novello et al., 2016; Haanen et al., 2017), and SITC (Puzanov et al., 2017; Rini et al., 2019) guidelines support the use of ICIs for various cancer types, with specific indications.

Despite the growth of the clinical indications for ICIs, the attitudes and practices of cancer physicians and patients in China toward ICIs are not clear. Side effects and tumor microenvironment currently represent the biggest obstacles in using ICIs. For example, severe immune-related AEs (irAEs) have also been observed following treatment with ICIs (Das and Johnson, 2019). Besides, some preclinical studies have shown that the intestinal flora may significantly affect the efficacy of a therapy targeting PD-1/PD-L1 (Sivan et al., 2015). Recently, we conducted a national questionnaire survey (which covered thirty different provinces and autonomous regions) to investigate the use of PD-1/PD-L1 inhibitors by oncologists in China (Zhang et al., 2020) and found that increasing numbers of oncologists were interested in using PD-1/PD-L1 inhibitors. In this study, we further expanded our search by conducting a more extensive cross-sectional survey. The survey aimed to investigate the concept and practices regarding ICIs in cancer patients across China (including all provinces and cities in China). The results provide real-world data on the AEs of ICIs and the factors influencing the use of ICIs in China.

## Materials and Methods

### Study Design and Participants

This was a cross-sectional survey of Chinese cancer patients. The study was initiated by the Expert Committee on Immuno-Oncology of the Chinese Society of Clinical Oncology (CSCO-IO) and the Expert Committee on Patient Education of the Chinese Society of Clinical Oncology (CSCO-PE). The surveys were handled using a web-based survey platform between November 29, 2019, and December 21, 2019. The patients from the hospitals cooperating with the CSCO-IO and CSCO-PE were invited consecutively to participate, covering all provinces and cities in China.

The ethics committee of Xinqiao Hospital affiliated with the Army Medical University (2019-Research No.127-01), approved the study. The front page of the survey presented the study and the implication for the patients. All patients signed the online version of the informed consent form before filling in the survey.

To be included, the patients had to have been diagnosed with any type of cancer before the start of the survey. The family members were allowed to assist the patient in responding when they were unable to read or move. The patients who never knew about ICI therapy were excluded from the survey.

### Survey Design

The survey was developed according to our previous study (Zhang et al., 2020). It was compiled after discussion and modification by the expert committees of the CSCO-IO and CSCO-PE. The survey contained four parts: basic information, information-seeking behavior, attitude regarding ICIs, and practice of using ICIs.

### Data Collection

Patients were recruited using two methods: 1) recruiting respondents through online platforms of a third-party survey agency, or 2) inviting eligible patients in the hospital. The patients who received online invitations were provided with the survey web link. The patients who received offline invitations filled in the survey by scanning the QR code. The geographic location of the respondents was determined through the network IP address following the survey submission.

### Quality Control

Quality control questions were set up in the survey to exclude pharmaceutical company/scientific research company personnel, physicians, and other medical personnel with high knowledge of ICIs. The survey had to be entirely completed upon submission, with no missing items.

### Statistical Analysis

Statistical analysis was carried out using SPSS 20.0 for Windows (IBM Corp., Armonk, NY, United States). Descriptive statistics were used. Categorical data are presented as numbers and percentages.

## Results

### Characteristics of the Respondents

A total of 2,459 surveys were sent, and 1,937 qualified surveys were collected (response rate of 78.8%). Besides, 362 patients (18.7%) who never knew about ICIs were excluded. Finally, 1,575 patients (81.3%) were included in the analysis.

**FIGURE 1:**
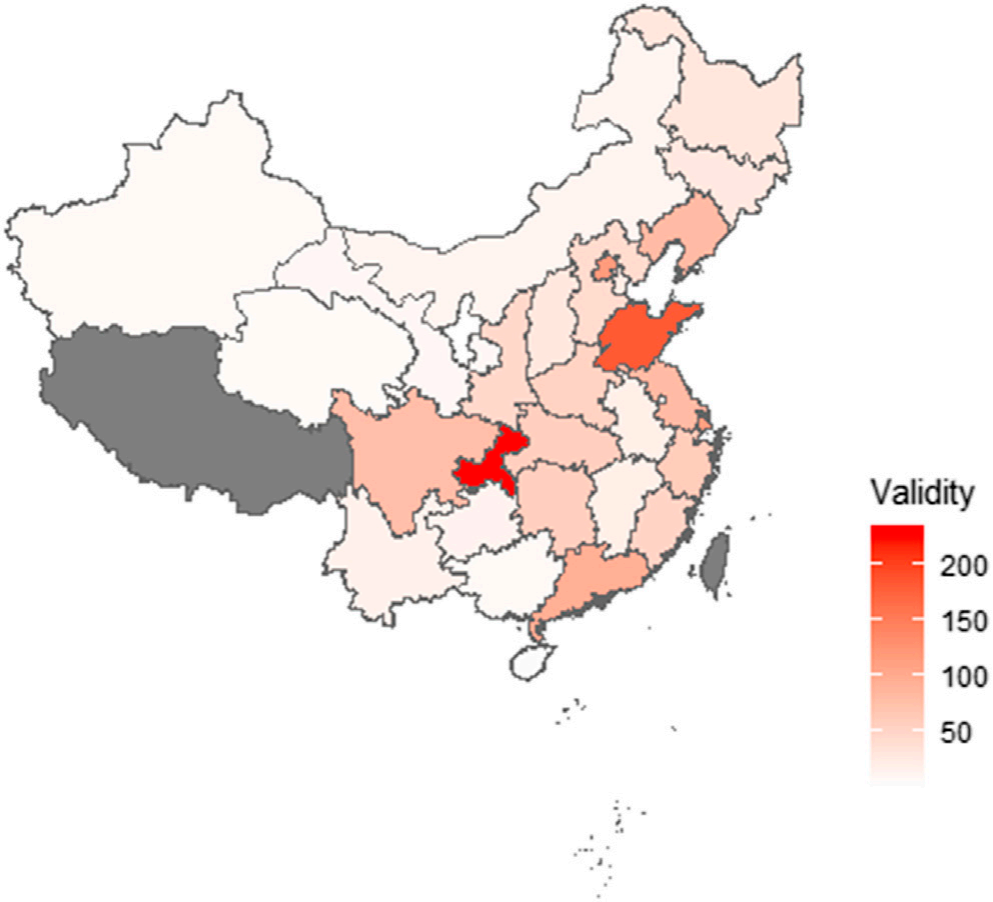
Province distribution of respondents. Valid surveys were collected from 30 provinces. The highest number of respondents were from the Chongqing area. Two respondents, who completed the survey outside China, are not shown.

Figure 1 shows the map of the province distribution of valid surveys. Valid surveys were collected from 30 provinces (Supplementary Table S1). The characteristics of the respondents are shown in Table 1. Most respondents were 41–60 years of age and undergraduate education (55.1%). Lung cancer was the primary tumor type (59.1%), followed by colorectal cancer (7.1%). The most known ICIs were pembrolizumab (55.3%), nivolumab (48.9%), sindilizumab (38.1%), and toripalimab (34.2%). The usage rate of ICIs was 32.4% (511/1,575). The remaining about two-thirds of patients and their families who had not used the ICIs had known about ICIs and were ready to choose ICIs.

**TABLE 1:** Characteristics of the survey respondents.

Characteristics	Total, n (%)
Age, years	
≤20	3 (0.2)
21–40	198 (12.6)
41–60	804 (51.0)
61–80	544 (34.5)
≥80	26 (1.7)
Education	
Junior high school and below	213 (13.5)
High school	342 (21.7)
Undergraduate	868 (55.1)
Master degree and above	152 (9.7)
Type of malignant tumor	
Age, years	
Lung cancer	931 (59.1)
Colorectal cancer	112 (7.1)
Breast cancer	71 (4.5)
Ovarian cancer	65 (4.1)
Liver cancer	62 (3.9)
Stomach cancer	47 (3.0)
Esophagus cancer	38 (2.4)
Cervical cancer	26 (1.7)
Melanoma	23 (1.5)
Pancreatic cancer	22 (1.4)
Thyroid cancer	15 (1.0)
Lymphoma	15 (1.0)
Prostatic cancer	10 (0.6)
Endometrial cancer	10 (0.6)
Bladder cancer	3 (0.2)
Other malignancies	125 (7.9)
Patients’ knowledge of ICIs	
Keytruda/pembrolizumab	871 (55.3)
Opdivo/nivolumab	770 (48.9)
Daboshu/sindilizumab	600 (38.1)
Tuoyi/toripalimab	538 (34.2)
Airika/camrelizumab	466 (29.6)
Tecentriq/atezolizumab	310 (19.7)
Age, years	
Tislelizumab	250 (15.9)
Yervoy/ipilimumab	213 (13.5)
Imfinzi/durvalumab	164 (10.4)
Bavencio/avelumab	117 (7.4)
Tremelimumab	76 (4.8)
Total usage rate of ICIs	511 (32.4)
Patient use of ICIs^a^	
Keytruda/pembrolizumab	174 (11.0)
Opdivo/nivolumab	10 8(6.9)
Tuoyi/toripalimab	93 (5.9)
Airika/camrelizumab	84 (5.3)
Daboshu/sindilizumab	7 7(4.9)
Tislelizumab	9 (0.6)
Tecentriq/atezolizumab	9 (0.6)
Yervoy/ipilimumab	7 (0.4)
Imfinzi/durvalumab	5 (0.3)
Bavencio/avelumab	4 (0.3)
Tremelimumab	3 (0.2)
Approach of access to ICIs^a^	
Pharmacy	322 (63.0)
Hospital	196 (38.4)
Charitable donation	72 (14.1)
Participation in clinical trials	50 (9.8)
Age, years	
Hong Kong, Macao, Taiwan, or overseas purchases	39 (7.6)
Wardmate approach (bought from others, transfer or gift)	60 (11.7)
Using place of ICIs^a^	
Hospital ward	428 (83.8)
Hospital outpatient	122 (23.9)
Community health service center	33 (6.5)
Private clinics	25 (4.9)
Home (medical home visit)	22 (4.3)
Pharmacy	7 (1.4)
Hong Kong, Macao, Taiwan, or overseas medical facilities	1 (0.2)

ICIs, immune checkpoint inhibitors.

^a^ The 511 patients who had been treated with ICIs were asked the following questions.

### Approaches and Preferences of Patients Related to ICIs

**FIGURE 2:**
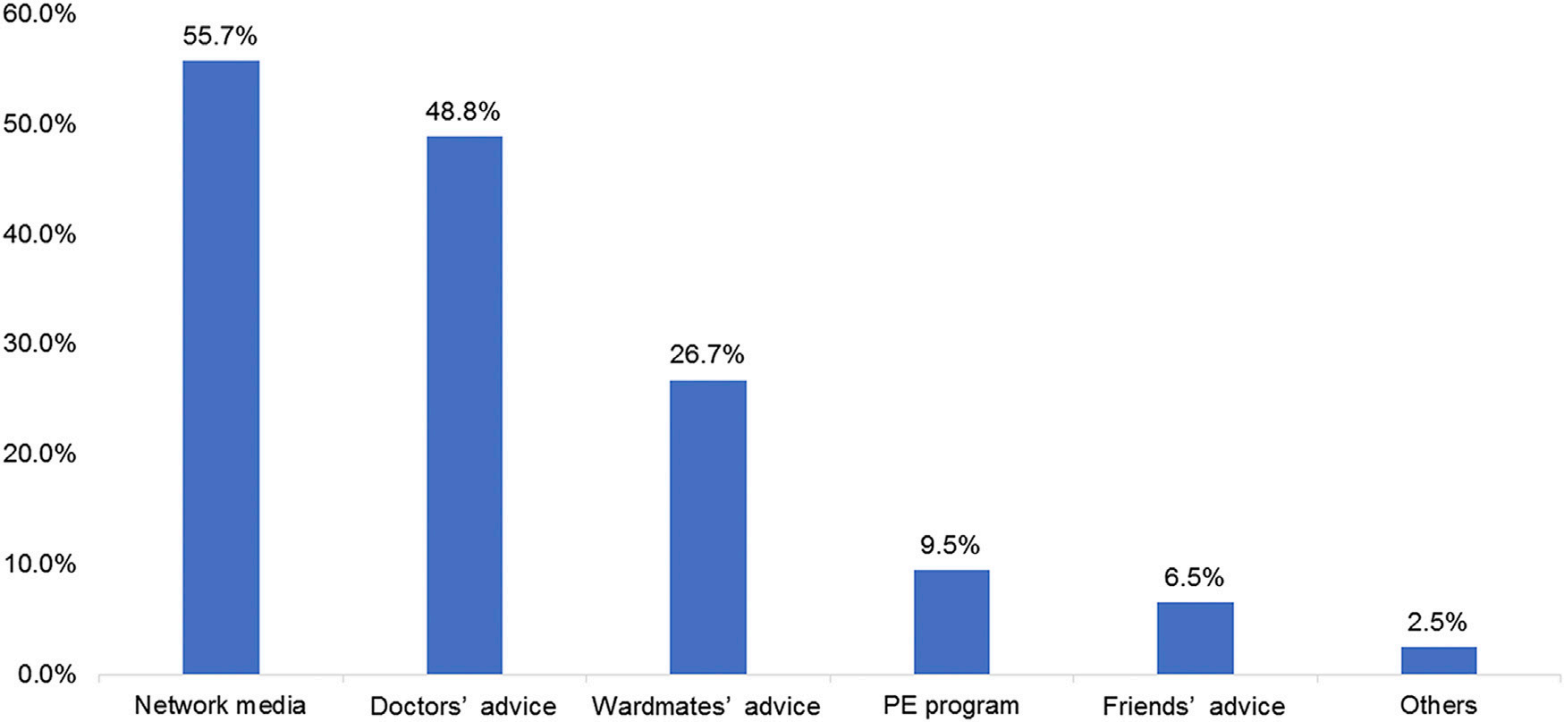
Frequency of the answers regarding the methods for learning about immune checkpoint inhibitors.

**FIGURE 3:**
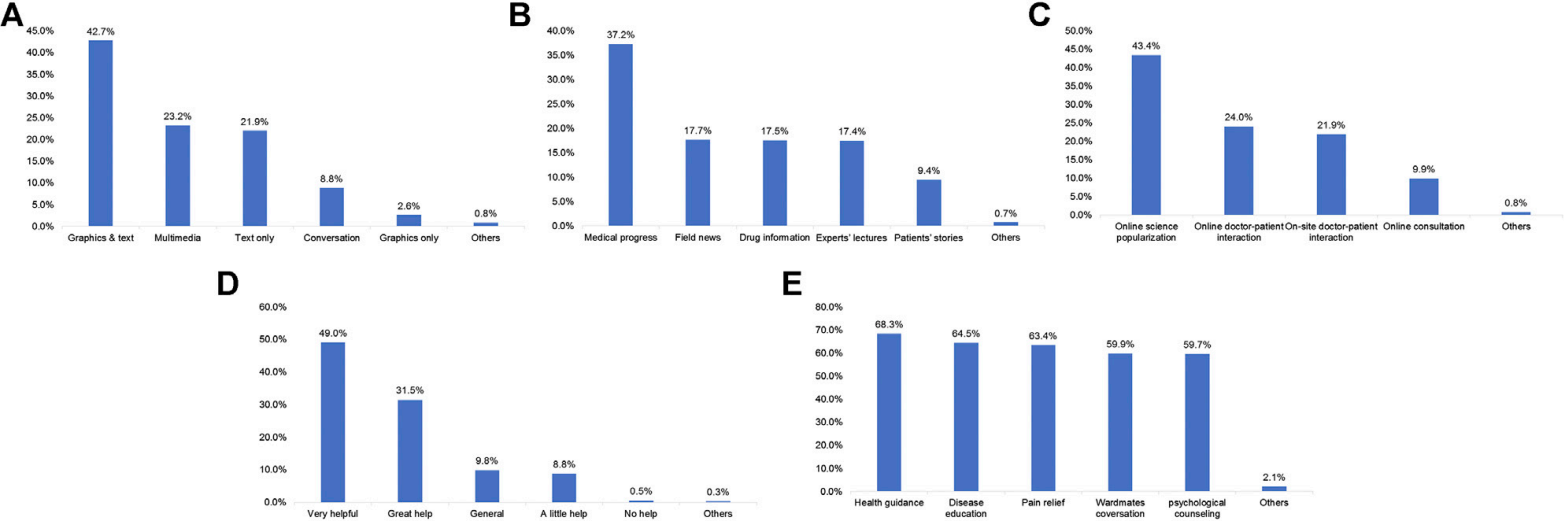
Frequencies of the approaches and preferences of the patients for acquiring immune checkpoint inhibitor treatment information. **(A)** Preferred types of written material for information about checkpoint inhibitors. **(B)** Preferred types of information about checkpoint inhibitors. **(C)** Preferred types of online and offline activities about checkpoint inhibitors. **(D)** Appreciation of the patients regarding wardmate group, patient education official accounts, APP, and rehabilitation organization about checkpoint inhibitors. **(E)** Patients’ need for oncology services.

Among the participants, 55.7% learned about ICIs from network media, 48.8% received doctors’ advice regarding ICIs therapy, 26.7% received ward mates’ advice, and 9.5 and 6.5% learned about ICIs from a patient education program and friends’ advice, respectively (Figure 2). The patients were most interested in graphics and text information (42.7%; Figure 3A). In terms of content type, the patients were most interested in medical progress (37.2%; Figure 3B). In terms of the type of activity, the patients were most interested in online science popularization (43.4%; Figure 3C). Besides, 49.0% of the patients thought that the existing information channels (wardmate groups, patient education official accounts, APP, and rehabilitation organization activity) were very helpful (Figure 3D). In terms of oncology services, the top three patients’ interests were health guidance (68.3%), disease education (64.5%), and cancer pain relief services (63.4%) (Figure 3F).

### Reasons for Using ICIs and Possible Concerns Regarding ICI Therapy

**FIGURE 4:**
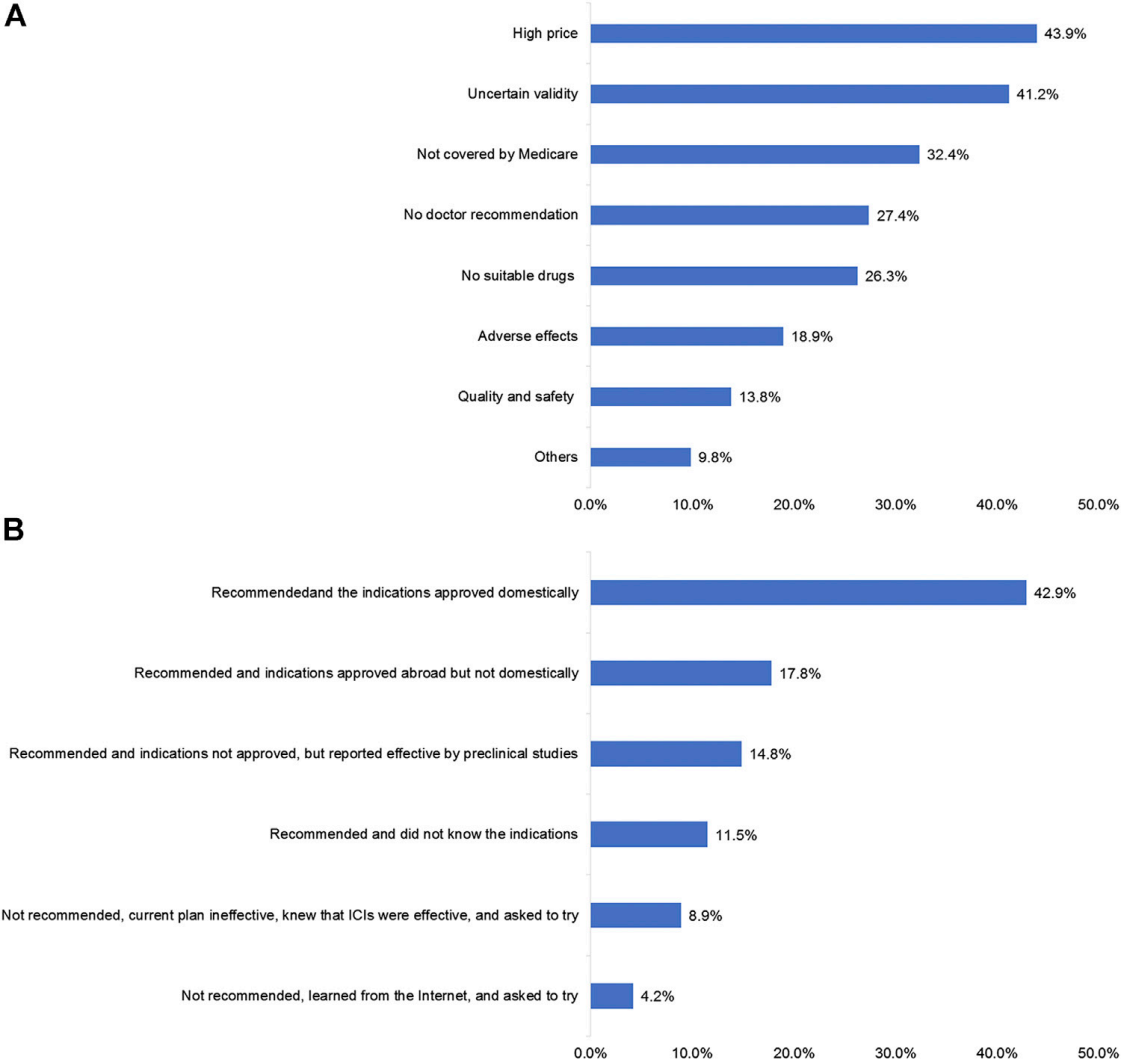
Factors influencing the use of ICIs in patients. **(A)** Patients’ reasons for not using immune checkpoint inhibitor (ICI) treatment. **(B)** Patients’ reasons for using ICI treatment.

High prices (43.9%), tuncertainty regarding drug efficacy (41.2%), and no reimbursement from medical insurance (32.4%) were the major factors that prevented the patients from using ICIs (Figure 4A). Domestic recommendations and the indications (42.9%), recommendations and indications approved abroad (17.8%), and recommendations and indications which were not approved but reported effective by preclinical studies (14.8%) were the driving factors for the patients to use ICIs (Figure 4B). The patients were most concerned about the therapeutic effect (40.3%), followed by the conditions for drug use (33.4%) (Supplementary Figure S1).

### Current Status of Drug Use: Efficacy and Adverse Effects

ICIs were considered effective in 9.0% of the patients whose tumor disappeared and 57.1% of the patients whose tumor volume reduced. ICIs were considered ineffective in 21.0% of the patients, who experienced no change in tumor volume, and in 12.9% of the patients whose tumor volume increased (Supplementary Figure S2).

Among the patients who received ICIs, 34.3% reported no AEs after ICIs. The most common AEs were immune-related dermatitis (36.3%), immune-related pneumonia (16.5%), and immune-related thyroid dysfunction (13.5%) (Supplementary Figure S3A). Among the patients who experienced AEs, 54.9% did not need to suspend treatment since they experienced mild AEs. In comparison, 18.5% required suspension or termination (14.6% with moderate AEs suspended the ICI treatment but resumed after irAE remission and 3.9% with severe AEs and discontinued ICI permanently) (Supplementary Figure S3B). Besides, when the patients were asked, “if you or your family initially used imported drugs with a good effect, would you consider switching to a domestic drug that has a lower price and approved related indications?”, 54.3% of patients said that they would consider a replacement, 17.3% said they would not, and 28.4% said that they would follow the doctor’s advice.

## Discussion

This national real-world survey investigated three main aspects (information seeking, attitude, and practice) related to ICIs in Chinese cancer patients. More than 80% of the patients were familiar with ICIs, and the most common ways of gaining relevant information (learning about ICIs therapy) were internet media, doctors, and ward mates. Pembrolizumab, nivolumab, and sindilizumab were the most commonly used ICIs. Furthermore, patients were most interested in learning about the existing graphics and texts and medical progress related to ICIs treatment. At the same time, the online popularization of science activity by doctors attracted the patients’ attention. More than 80% of the patients recognized the help of patient groups, patient education official accounts or APP, and rehabilitation organizations, and patients had a strong demand for various types of cancer services. Moreover, approximately two-thirds of the patients have never been treated with ICIs, primarily due to economic reasons or uncertainty about drug effectiveness. Similar data were reported by Haslam and Prasad (2019). They found that 43.6% of cancer patients in the United States were eligible for ICIs in 2018 (Haslam and Prasad, 2019); yet, their study did not examine the number of patients treated with ICIs.

Our data indicated that doctors’ recommendations were the most important reason for patients to use ICIs. More than 50% of the patients used pembrolizumab or nivolumab. The efficacy, effective duration, conditions of use of the drug, and types of tests required during therapy were the most important concerns.

Immune-related dermatitis was the most common irAE. In addition, 50% of the patients reported that the degree of the AEs was mild, and there was no need to suspend the ICIs. This was not consistent with a previous study investigating the use of ICIs in advanced lung cancer and found a high rate of ICI discontinuation due to AEs (Muchnik et al., 2019). Suresh *et al.* indicated that the rate of ICI-induced pneumonitis in lung cancer patients could be as high as 7–19% (Suresh et al., 2018). A study reported a rate of 33% of pneumonitis with any symptoms (grade ≥2) (Saito et al., 2020). A meta-analysis showed that the pooled rates of grades 2, 3, 4, and 5 pneumonitis were 17.8, 7.9, and 14%, respectively (Dawe et al., 2016). The cancer type, cancer stage, ECOG, and previous treatments might be important factors in the patients’ response to ICIs. Our data indicated that the patients paid more attention to the indications and price when considering ICIs therapy, and less concern about the origin of the drug (domestic or imported drug), while only less than 20% of patients were reluctant to change to domestic drugs after the approval of domestic drugs. Finally, pharmacies were the most important way for patients to obtain ICIs, followed by hospitals. The vast majority of patients received drug infusions at hospitals.

These results present the real-world situation of the use of ICIs in China, according to the patients’ perspective. Unfortunately, considering different research settings, it was impossible to compare the results from this survey with the results from clinical trials. Real-world studies about the attitude and practice toward ICIs are rare. Notably, many patients receive ICIs outside of the approved indications in China. As ICIs in China are just in the preliminary stage of obtaining their clinical approval, the clinical use of ICIs might lag behind the global ones. In addition, the patients learn about ICIs from the internet and the news, and many of them are willing to receive ICIs beyond the approved indications.

This study has some limitations. First, there were no restrictions on the type of tumor, which might reflect the real situation more comprehensively, but introduce a higher heterogeneity in the results. Second, this was a cross-sectional study without follow-up. The study was designed to investigate the attitudes and practices, and no follow-up was required. Third, the patients were concentrated in some provinces, possibly because the current ICIs are still not being used in some provinces with poorer economic conditions, leading to biases in the reported results. Fourth, the patients who never heard of ICIs were excluded, which introduced some bias. Fifth, the treatment pattern of ICIs was not collected in the survey, including setting of lines, combination medication and off-label use. Finally, the rates of severe AEs and treatment discontinuation due to AEs were smaller than those reported in previous studies (Suresh et al., 2018; Muchnik et al., 2019). It is possible that there was a response bias. Of note, a possible correlation between AE severity and response is suspected (Palmieri and Carlino, 2018).

## Conclusion

Cancer patients in China have a preliminary understanding of emerging ICIs through physicians’ direct education or the internet. Besides, patients are concerned about medical progress and the doctors’ popularization of science. They also think that the educational information provided by the media was helpful. Uncertainty about the efficacy and economic factors are the main obstacles in using ICIs. For patients who received ICIs, the conditions of use of drugs are the most critical concerns. More than 50% of the patients reported that the treatment is effective. Although the AEs of ICIs are relatively common, most of them are mild and moderate.

## Data Availability

The original contributions presented in the study are included in the article/Supplementary Material, further inquiries can be directed to the corresponding author.
