# Supplementary material for: Attitudes and Practices of Immune Checkpoint Inhibitors in Chinese Patients With Cancer: A National Cross-Sectional Survey
Source: Front Pharmacol. 2021 Mar 22;12:583126. doi: 10.3389/fphar.2021.583126 (PMC8025873; doi:10.3389/fphar.2021.583126)
Supplement: Supplementary file 1 [file Image1.tif]

Frontiers | Attitudes and practices of immune checkpoint inhibitors in Chinese patients with cancer: A national cross-sectional survey | Pharmacology


- About
- Journals
- Research Topics
- Articles
- More

Submit

My Frontiers

Office

- TSOF
  - TSOF
  - Article Production

Typesetter 3

frontiersproduction@tnq.co.in

- Profile
- Settings & Privacy
- Help Center
- Logout

Submit

**Impact Factor 4.225** | **CiteScore 5.0**More on impact ›

|  |  |
| --- | --- |
| Frontiers in Pharmacology | Pharmacology of Anti-Cancer Drugs |

Toggle navigation


Section


- (current)Section
- About
- Articles
- Research topics
- For authors 
  - Why submit?
  - Fees
  - Article types
  - Author guidelines
  - Review guidelines
  - Submission checklist
  - Contact editorial office
  - Submit your manuscript
- Editorial board

- *Article alerts*

##### This article is part of the Research Topic

Targeted Immunotherapy for Cancer
View all
17
Articles

Articles


**Suggest a Research Topic >**

- 48
  total views

 View Article Impact

**Suggest a Research Topic >**

##### SHARE ON

- Facebook

  0
- Twitter

  0
- LinkedIn

  0
- AddThis

  New


## Original Research ARTICLE

Front. Pharmacol.
| doi: 10.3389/fphar.2021.583126

# Attitudes and practices of immune checkpoint inhibitors in Chinese patients with cancer: A national cross-sectional survey Provisionally accepted The final, formatted version of the article will be published soon. **Notify me**

Luping Zhang1,  Jun Wang2,  Bicheng Zhang3,  Qian Chu4,  Chunxia Su5, Hao Wu6, 
Saiqi Wang7, 
Baocheng Wang8, 
Yongmei Yin6, 
Bo Zhu1 and 
 Jianguo Sun1\*

- 1Xinqiao Hospital, China
- 2Qianfoshan Hospital, Shandong University, China
- 3Renmin Hospital of Wuhan University, China
- 4Tongji Hospital, Tongji Medical College, Huazhong University of Science and Technology, China
- 5Shanghai Pulmonary Hospital, School of Medicine, Tongji University, China
- 6First Affiliated Hospital, Nanjing Medical University, China
- 7Henan Provincial Cancer Hospital, China
- 8960th Hospital of the PLA, China

Immune-checkpoint inhibitors (ICIs) are revolutionizing the field of immuno-oncology. Side effects and tumor microenvironment currently represent the most significant obstacles to using ICIs. In this study, we conducted an extensive cross-sectional survey to investigate the concept and practices regarding the use of ICIs in cancer patients in China. The results provide real-world data on the adverse events (AEs) of ICIs and the factors influencing the use of ICIs. This survey was developed by the Expert Committee on Immuno-Oncology of the Chinese Society of Clinical Oncology (CSCO-IO) and the Expert Committee on Patient Education of the Chinese Society of Clinical Oncology (CSCO-PE). The surveys were distributed using a web-based platform between 11-29-2019 and 12-21-2019. A total of 1575 patients were included. High costs (43.9%), uncertainty about drug efficacy (41.2%), and no reimbursement from medical insurance (32.4%) were the factors that prevented the patients from using ICIs. The patients were most concerned about the onset time or effective duration of ICIs (40.3%), followed by the indications of ICIs and pre-use evaluation (33.4%). Moreover, 9.0%, 57.1%, 21.0%, and 12.9% of the patients reported tumor disappearance, tumor volume reduction, no change in tumor volume, and increased tumor volume. Among the patients who received ICIs, 65.7% reported immune-related AEs (irAEs); 96.1% reported mild-to-moderate irAEs. Cancer patients in China had a preliminary understanding of ICIs. Yet, the number of patients treated with ICIs was small.

Keywords: 
Immunotherapy, adverse effects, Attitude, Practice, Survey

Received: 17 Jul 2020;
Accepted: 26 Jan 2021.

Copyright: © 2021 Zhang, Wang, Zhang, Chu, Su, Wu, Wang, Wang, Yin, Zhu and Sun. This is an open-access article distributed under the terms of the Creative Commons Attribution License (CC BY). The use, distribution or reproduction in other forums is permitted, provided the original author(s) and the copyright owner(s) are credited and that the original publication in this journal is cited, in accordance with accepted academic practice. No use, distribution or reproduction is permitted which does not comply with these terms.

\* Correspondence: 
Mx. Jianguo Sun, Xinqiao Hospital, Chongqing, China, sunjg09@aliyun.com

Write a comment...

Add

##### COMMENTARY

##### ORIGINAL ARTICLE

##### People also looked at

**Suggest a Research Topic >**

×

#### Supplementary Material

  

There is no supplementary material currently available for this article

Loading supplemental data...

  

|  | File Name |  |
| --- | --- | --- |
|  | Table 1.DOCX |  |
|  | Image 1.TIF |  |
|  | Image 2.TIF |  |
|  | Image 3.TIF |  |

  

Close

- About Frontiers
- Institutional Membership
- Books
- News
- Frontiers' social media
- Contact
- Careers
- Submit
- Newsletter
- Help Center
- Terms & Conditions
- Privacy Policy

© 2007 - 2021 Frontiers Media S.A. All Rights Reserved

### Privacy Preference Center

Our website uses cookies that are necessary for its operation. Additional cookies are only used with your consent. These cookies are used to store and access information such as the characteristics of your device as well as certain personal data (IP address, navigation usage, geolocation data) and we process them to analyse the traffic on our website in order to provide you a better user experience, evaluate the efficiency of our communications and to personalise content to your interests. Some cookies are placed by third-party companies with which we work to deliver relevant ads on social media and the internet. Click on the different categories' headings to change your cookie preferences. Click on "More Information" if you wish to learn more about how data is collected and shared.
More information

### Manage Consent Preferences

#### Strictly Necessary Cookies

Always Active

These cookies are necessary for the website to function and cannot be switched off in our systems. They are usually only set in response to actions made by you which amount to a request for services, such as setting your privacy preferences, logging in or filling in forms. You can set your browser to block or alert you about these cookies, but some parts of the site will not then work. These cookies do not store any personally identifiable information.

#### Analytics Cookies

Analytics Cookies

These cookies allow us to count visits and traffic sources so we can measure and improve the performance of our site. They help us analyse which pages are the most and least popular and see how visitors move around the site.    All information these cookies collect is aggregated and therefore anonymous.

#### Functional Cookies

Functional Cookies

These cookies enable the website to provide enhanced functionality and personalisation. They may be set by us or by third party providers whose services we have added to our pages. If you do not allow these cookies then some or all of these services may not function properly.

#### Advertising Cookies

Advertising Cookies

These cookies may be set through our site by our advertising partners. They may be used by those companies to build a profile of your interests and show you relevant adverts on other sites.    They do not store directly personal information, but are based on uniquely identifying your browser and internet device. If you do not allow these cookies, you will experience less targeted advertising.

### Back Button Performance Cookies

Vendor Search  Search Icon

Filter Icon

Clear

checkbox label label

Apply Cancel

Consent Leg.Interest

checkbox label label

checkbox label label

checkbox label label

Confirm My Choices
