# Supplementary material for: Attitudes and Practices of Immune Checkpoint Inhibitors in Chinese Patients With Cancer: A National Cross-Sectional Survey
Source: Front Pharmacol. 2021 Mar 22;12:583126. doi: 10.3389/fphar.2021.583126 (PMC8025873; doi:10.3389/fphar.2021.583126)
Supplement: Supplementary file 4 [file table1.docx]

**Table S1.** Province distribution of the respondents

| Region | Number of respondents |
| --- | --- |
| Anhui | 19 |
| Beijing | 117 |
| Fujian | 51 |
| Gansu | 9 |
| Guangdong | 96 |
| Guangxi | 7 |
| Guizhou | 14 |
| Hainan | 2 |
| Hebei | 43 |
| Henan | 54 |
| Heilongjiang | 30 |
| Hubei | 58 |
| Hunan | 58 |
| Jilin | 27 |
| Jiangsu | 82 |
| Jiangxi | 19 |
| Liaoning | 82 |
| Inner Mongolia | 13 |
| Ningxia | 1 |
| Qinghai | 5 |
| Shandong | 183 |
| Shanxi | 29 |
| Shaanxi | 46 |
| Shanghai | 113 |
| Sichuan | 78 |
| Tianjin | 23 |
| Xinjiang | 7 |
| Yunnan | 17 |
| Zhejiang | 61 |
| Chongqing | 229 |
| Outside China | 2 |
| Total | 1575 |
